# Comparison of DTI analysis methods for clinical research: influence of pre-processing and tract selection methods

**DOI:** 10.1186/s41747-018-0066-1

**Published:** 2018-11-14

**Authors:** Volker Ressel, Hubertus J. A. van Hedel, Ianina Scheer, Ruth O’Gorman Tuura

**Affiliations:** 1 Centre MR-Research, University Children’s Hospital, Zurich, Switzerland; 2 Rehabilitation Centre, University Children’s Hospital, Mühlebergstrasse 104, CH-8910 Affoltern am Albis, Switzerland; 3 Children’s Research Center, University Children’s Hospital, Zurich, Switzerland; 4 Diagnostic Imaging, University Children’s Hospital, Zurich, Switzerland

**Keywords:** Anisotropy, Brain injuries, Diffusion tensor imaging, Image processing (computer-assisted), Paediatrics

## Abstract

**Background:**

The primary aim was to compare fractional anisotropy (FA) values derived with different diffusion tensor imaging (DTI) analysis approaches (atlas-based, streamline tractography, and combined). A secondary aim was to compare FA values and number of tracts (NT) with the clinical motor outcome quantified by the functional independence measure for children (WeeFIM).

**Methods:**

Thirty-nine DTI datasets of children with acquired brain injury were analysed. Regions of interest for the ipsilesional corticospinal tract were defined and mean FA and NT were calculated. We evaluated FA values with Spearman correlation, the Friedman and Wilcoxon tests, and Bland-Altman analysis. DTI values were compared to WeeFIM values by non-parametric partial correlation and accuracy was assessed by receiver operating characteristics analysis.

**Results:**

The FA values from all approaches correlated significantly with each other (*p* < 0.001). However, the FA values from streamline tractography were significantly higher (mean ± standard deviation (SD), 0.52 ± 0.08) than those from the atlas-based (0.42 ± 0.11) or the combined approach (0.41 ± 0.11) (*p* < 0.001 for both). FA and NT values correlated significantly with WeeFIM values (atlas-based FA, partial correlation coefficient (ρ) = 0.545, *p* = 0.001; streamline FA, ρ = 0.505, *p* = 0.002; NT, ρ = 0.434, *p* = 0.008; combined FA, ρ = 0.611, *p* < 0.001). FA of the atlas-based approach (sensitivity 90%, specificity 67%, area under the curve 0.82) and the combined approach (87%, 67%, 0.82), provided the highest predictive accuracy for outcome compared to FA (70%, 67%, 0.67) and NT (50%, 100%, 0.79, respectively) of the streamline approach.

**Conclusion:**

FA values from streamline tractography were higher than those from the atlas-based and combined approach. The atlas-based and combined approach offer the best predictive accuracy for motor outcome, although both atlas-based and streamline tractography approaches provide significant predictors of clinical outcome.

## Key points

- Ipsilesional FA values differed according to the approach used for tract selection
- However, FA derived using both atlas-based and streamline approaches correlated with outcome
- Atlas-based and combined approach provide better prediction for outcome than streamline tractography
- A combined approach with additional motion correction could improve prediction of rehabilitation outcome

## Background

Diffusion tensor imaging (DTI) is a noninvasive method for investigating cerebral microstructure *in vivo* in both research and clinical settings. Based on the principle that the motion of water along the axis of a bipolar magnetic field gradient will induce a phase change causing signal attenuation in magnetic resonance imaging (MRI), diffusion-weighted images can be collected. These images are sensitised to microscopic, orientation-dependent motion of water molecules. Since water diffusion occurs within and outside cellular structures, the degree of water diffusion in the brain depends on the local cellular microstructure. By collecting multiple diffusion-weighted images with different encoding gradient directions, it is possible to characterise the three-dimensional pattern of water diffusion with a diffusion tensor model, incorporating information about the directionality and the magnitude of diffusion at each point in the brain [1, 2]. The orientation dependence of water diffusion measured with DTI can be quantified with diffusion anisotropy measures calculated from the diffusion tensor such as the fractional anisotropy (FA), which varies in magnitude from a value of 0 (indicating that proton spins in water can diffuse randomly in any direction) to a value of 1 (indicating that water diffusion is restricted only to one direction). DTI metrics can also be used to characterise the structural connectivity between brain regions, and are sensitive to developmental (*e.g.*, myelination) and pathological (*e.g.*, ischaemic, inflammatory) processes affecting the microstructure of white matter tracts. DTI is, therefore, widely used for many clinical applications [3] and has previously been used to predict motor outcome in children with acquired brain injuries (ABI) [4].

Different analysis approaches have been developed to analyse DTI data. The standard analysis steps include pre-processing of the data (*e.g.*, eddy current correction), tensor estimation, definition of regions of interest (ROI), and tractography. Optionally, for group analyses, a normalisation step can be applied followed by statistical inference testing, as implemented in the tract-based spatial statistics pipeline [5]. Soares et al. [6] published a hitchhiker’s guide of relevant software and approaches used in clinical and research studies, suggesting a possible pipeline for DTI data analysis. A list of common pitfalls has been reported by Jones and Cercignani [7]. However, only a few studies have compared the test-retest reproducibility of DTI metrics derived with different analysis approaches, like tract-based and cross-sectional methods [8], or provided practical guidance for the application of different techniques (ROI, tractography, voxel-based analysis) [9]. Comparisons of clinical or research DTI data and results between analysis approaches remain scarce.

In this study, we selected two commonly used analysis approaches, namely an atlas-based approach and a streamline tractography approach, implemented in two popular analysis packages (FMRIB software library [FSL] and ExploreDTI). Both methods are freely available and perform all the basic steps necessary to analyse DTI data, but differ on many technical points. Therefore, the first aim of this study was to compare FA values derived with different DTI analysis approaches taking the pre-processing pipelines, tract selection methods, and the sensitivity of analysis approaches into account in their standard implementation. The second aim was to compare the accuracy of different approaches for predicting motor outcome in a clinical-research DTI dataset of children and adolescents with ABI, where the clinical motor outcome was quantified by the functional independence measure for children (WeeFIM). The advantages and disadvantages of each method, the relative merits of a combined approach, and the relevance of each method for clinical application are also discussed.

## Methods

### Patient population

Thirty-nine DTI datasets of children (20 male, 19 female) with ABI were used for this study (Table 1). The patients ranged in age from 1.1 to 19.4 years (mean 9.1 years, standard deviation [SD] 4.6 years) and were referred for MRI at the University Children’s Hospital (Zurich) in 2010–2016 for stroke (*n* = 20) or traumatic brain injury (TBI) (*n* = 19). All patients completed a standard clinical 3 T MRI examination (Signa HD.xt/MR750, General Electric Healthcare, Milwaukee, WI, USA) including DTI at 1–288 days after injury (median = 28 days), before admission to the rehabilitation centre. Ethical approval was received from the local ethical committee and written informed consent was obtained from all participants and/or their parents for the inclusion of their data in this retrospective study.

**Table 1:** Patients and diffusion tensor imaging characteristics

Patients	Characteristics				MRI	
	Gender	Age at rehabilitation (years)	Time in rehabilitation (days)	Time after Injury (days)	Main lesion side	DTI protocol
Stroke (*n* = 20)						
1	f	19.39	11	288	left	21 Dir
2	f	15.64	32	1	right	21 Dir
3	f	14.29	144	158	left	21 Dir
4	f	4.14	289	4	left	21 Dir
5	f	3.47	23	21	left	21 Dir
6	f	2.51	218	121	left	35 Dir
7	f	5.95	24	9	right	35 Dir
8	f	13.92	129	12	left	35 Dir
9	m	11.41	38	1	right	21 Dir
10	m	16.39	24	54	left	21 Dir
11	m	2.83	121	31	right	21 Dir
12	m	14.13	220	46	left	21 Dir
13	m	3.13	562	28	right	21 Dir
14	m	10.05	78	252	left	21 Dir
15	m	5.16	43	24	right	35 Dir
30	f	10.90	129	7	left	35 Dir
31	f	6.03	163	1	right	35 Dir
32	m	15.42	59	135	right	35 Dir
33	m	11.41	78	8	right	35 Dir
34	m	7.08	254	3	right	35 Dir
Average		9.66	131.95	60.20		
SD		5.33	131.51	85.83		
TBI (*n* = 19)						
16	f	10.81	50	50	left	21 Dir
17	f	11.92	144	6	left	21 Dir
18	f	9.48	118	4	left	35 Dir
19	f	9.27	189	3	right	35 Dir
20	f	7.75	135	92	right	35 Dir
21	f	7.15	25	14	right	35 Dir
22	m	1.12	175	104	right	21 Dir
23	m	1.30	211	108	left	21 Dir
24	m	10.43	175	86	left	35 Dir
25	m	7.60	22	5	right	35 Dir
26	m	13.25	365	81	left	35 Dir
27	m	10.19	44	1	left	35 Dir
28	m	10.41	106	144	left	35 Dir
29	m	6.26	34	2	right	35 Dir
35	f	13.28	52	9	right	35 Dir
36	f	15.54	105	2	left	35 Dir
37	f	5.91	159	151	left	35 Dir
38	m	5.66	161	17	right	35 Dir
39	m	5.43	32	129	left	35 Dir
Average		8.57	121.16	53.05		
SD		3.82	85.83	55.16		
Total (*n* = 39)						
Average		9.13	126.69	56.72		
SD		4.63	110.30	71.67		

*Dir* directions, *DTI* diffusion tensor imaging, *f* female, *m* male, *SD* standard deviation, *TBI* traumatic brain injury

### MRI measurement

The MRI protocol included anatomical T1-weighted images (echo time (TE) 18 ms, repetition time (TR) 600 ms, slice thickness 4 mm, voxel resolution 1 × 1 × 4 mm^3^) and T2-weighted fast spin-echo images (TE 112 ms, TR 5000–6000 ms, slice thickness 3 mm, voxel resolution 0.5 × 0.5 × 3 mm^3^). DTI images were collected with a pulsed gradient-spin-echo sequence with an echo-planar imaging (EPI) readout (field of view, 240 mm, mean TE 89.5 ms, TE from 76.5 to 98.4 ms, TR 5975 ms, slice thickness 3 mm, acquisition matrix 128 × 128, reconstructed matrix size 256 × 256, reconstructed voxel resolution 0.94 × 0.94 × 3 mm^3^). Patients studied from 2010 to 2012 (*n* = 15) were scanned with a DTI protocol incorporating 21 gradient directions, while patients scanned from 2013 to 2016 (*n* = 24) were scanned with a protocol incorporating 35 gradient directions (see Table 1).

### Pre-processing

**Fig. 1:**
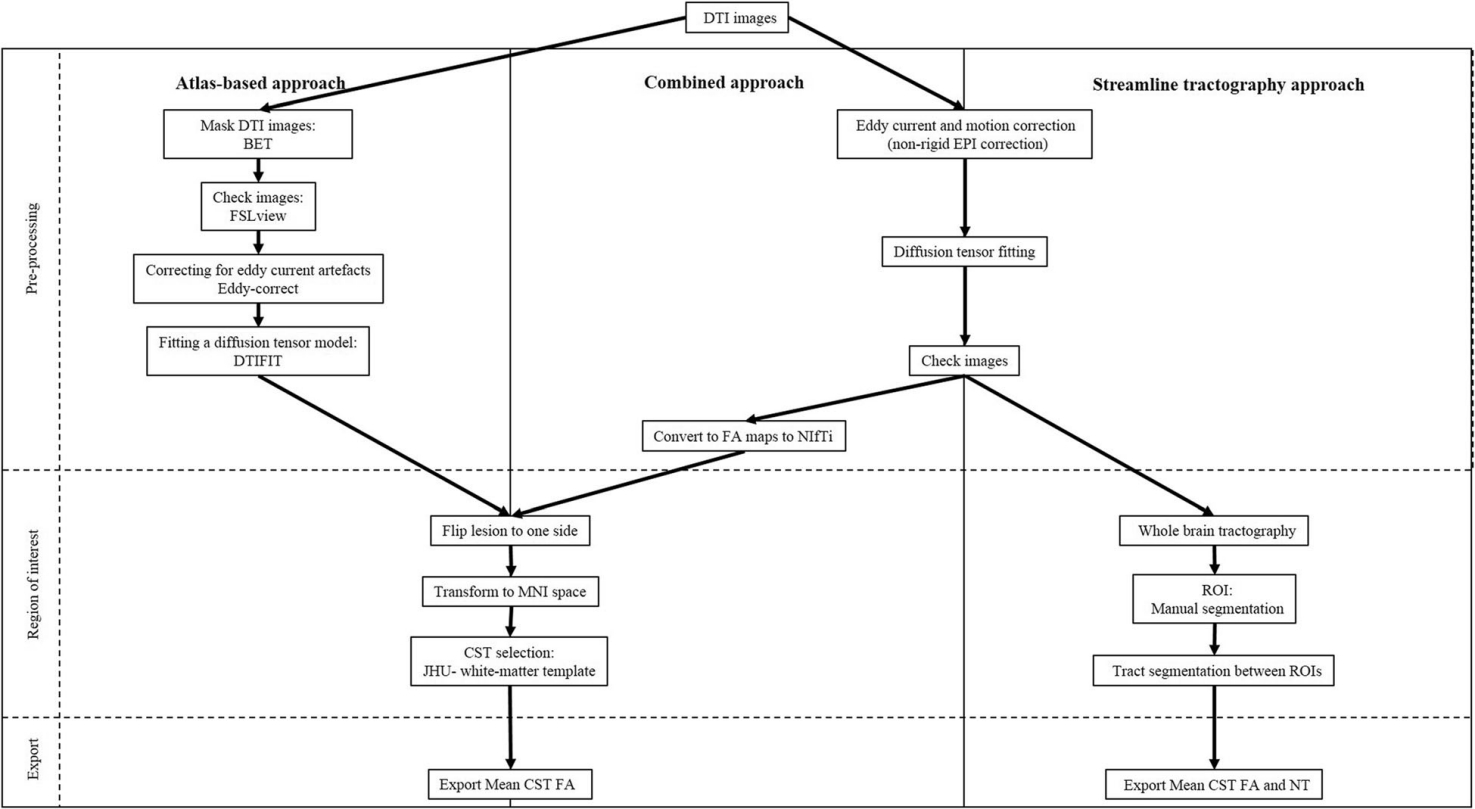
Workflow for the data processing in the different approaches. *BET*, brain extraction tool; *CST*, corticospinal-tract; *DTI*, diffusion tensor imaging; *EPI*, echo planar imaging; *FA*, fractional anisotropy; *FSL*, FMRIB software library; *JHU*, Johns Hopkins University; *MNI*, Montreal Neurological Institute; *NIfTi*, Neuroimaging Informatics Technology Initiative; *NT*, number of tracts; *ROI*, region of interest

Data were processed using two analysis approaches, an atlas-based method utilising tools from the FMRIB software library (FSL, Oxford, UK [5, 10]) and a streamline tractography approach as implemented in ExploreDTI [11] (Fig. 1).

For the atlas-based analysis the preprocessing followed the methods described in a previous study [4]. In summary, the pre-processing included skull stripping and masking the DTI images with the brain extraction tool, correcting for eddy current artefacts with eddy-correct, and fitting a diffusion tensor model at each voxel with DTIFIT toolbox. Images from patients with right-sided lesions were flipped (*n* = 18) so that all lesions were located on the left side for subsequent analysis steps. The fractional anisotropy (FA) maps for each patient were then normalised to Montreal Neurological Institute (MNI) space using FMRIB’s Linear Image Registration Tool [12]. For the analysis of the motor tracts the ipsilesional corticospinal tracts (CST) were defined using the Johns Hopkins University white-matter template. Data for the mean FA were exported for further statistical analysis.

**Fig. 2:**
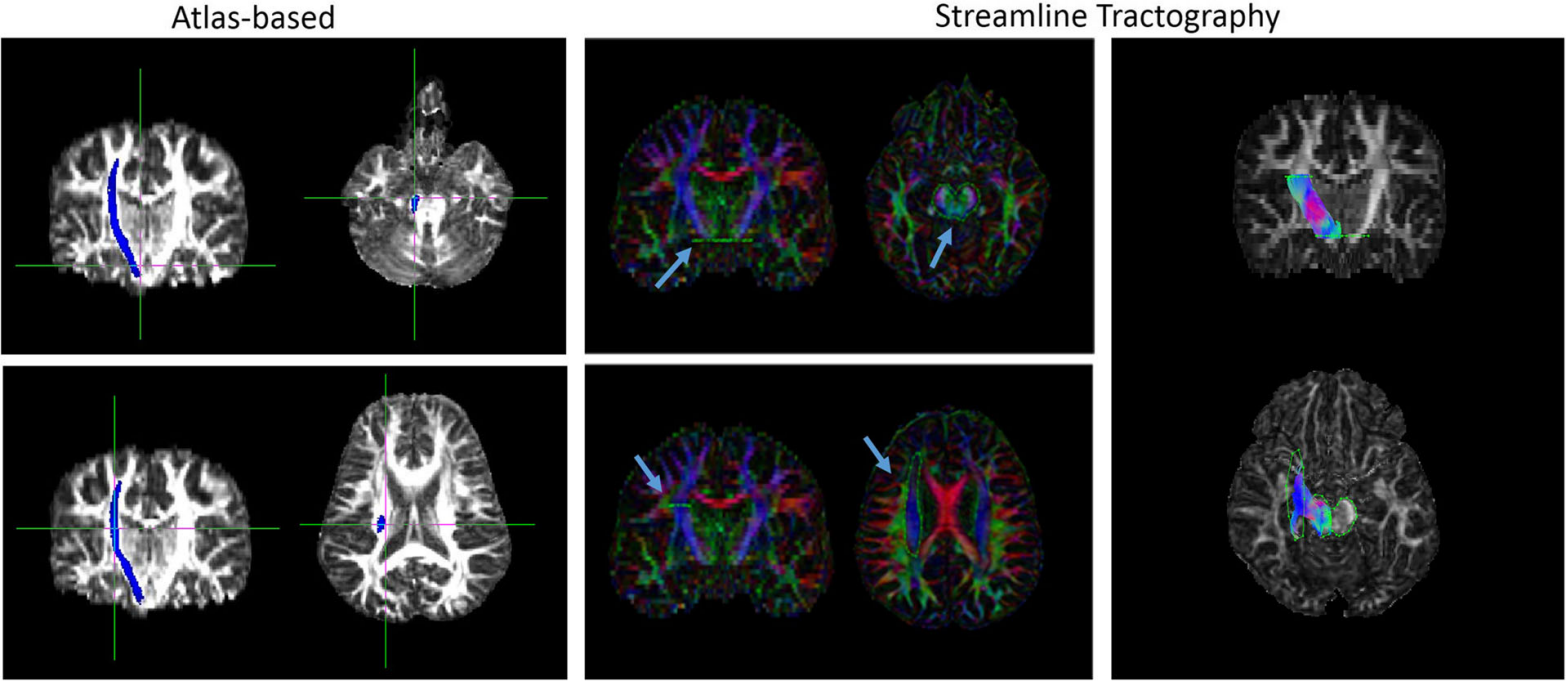
Example of regions of interest used for the atlas-based (FMRIB software library [FSL] software) and streamline tractography (ExploreDTI software) approach. The corticospinal-tracts were defined for the FSL software automatically from the Johns Hopkins University white-matter template and for the ExploreDTI software manually between the superior corona radiata and the cerebral peduncles. The corticospinal-tract is marked in blue (with a blue arrow), respectively, for both the atlas-based and streamline tractography methods

For the streamline tractography, pre-processing included eddy current and motion correction (non-rigid EPI correction), and whole brain tractography (standard settings). Subsequently, the CST was segmented by manually defining seed and target ROIs according to Nagae et al. [13]. The superior ROI was defined on an axial slice to include the superior corona radiata, and the inferior ROI was defined on an axial slice to include the cerebral peduncles. CST were then calculated from the whole-brain tractography, using a tract segment defined by the manually defined ROIs (Fig. 2). Data on the mean FA and number of tracts (NT) were then exported for further statistical analysis.

Additionally, to compare the effect of differences in pre-processing on the sensitivity to predict motor outcome, pre-processed FA maps from the whole-brain streamline tractography analysis were transformed into the MNI space where the mean FA of the CST were calculated using the Johns Hopkins University white-matter template for tract selection. The mean FA for each tract was then exported for further statistical analysis. We referred to this combination of methods as the “combined approach” (see Fig. 1).

### Functional outcome

Functional outcome was assessed as previously described [4]. In summary, the functional independence measure for children (WeeFIM) is a standard measurement used at the rehabilitation centre and was evaluated at discharge from the rehabilitation centre by professional caregivers and nurses [14]. Due to the multidimensionality of the WeeFIM and the focus of this retrospective study on functional motor outcome, we performed analyses only for the functional “motor” items in the self-care domain (i.e., without the items bladder and bowel, because these are also under autonomic control), and the mobility domain, excluding the cognition domain (Table 2).

**Table 2:** Motor functional independence measure for children scores for each patient

Patient	WeeFIM at discharge of rehabiitation		
	Self-care	Mobility	Total
1	42	35	77
2	42	35	77
3	37	35	72
4	6	5	11
5	23	32	55
6	8	5	13
7	12	14	26
8	42	35	77
9	40	35	75
10	41	35	76
11	11	18	29
12	39	35	74
13	9	8	17
14	15	22	37
15	12	6	18
16	42	35	77
17	42	31	73
18	41	34	75
19	39	34	73
20	42	33	75
21	38	33	71
22	8	7	15
23	8	10	18
24	42	35	77
25	38	35	73
26	42	35	77
27	42	35	77
28	40	35	75
29	36	35	71
30	41	35	76
31	29	32	61
32	30	27	57
33	42	35	77
34	40	35	75
35	42	35	77
36	42	35	77
37	31	32	63
38	21	27	48
39	32	34	66
Average	35.51	28.43	59.95
SD	13.09	10.49	23.22

Please note, self-care values without the items bladder and bowel

*SD* standard deviation, *WeeFIM* functional independence measure for children

Patients were classified as having a good outcome if their age-normalised WeeFIM scores at discharge were greater than 85% of the age-normalised maximum value. This threshold was based on the scores of each WeeFIM item. A healthy 7 year-old-child should score the maximal score of 7 on each of the 18 items. As only scores of 6 and 7 indicate independence (6/7 ≈ 85%), 85% of the maximal score should indicate independence, and “good” versus “poor” outcome would, therefore, reflect independent versus dependent outcome. Since children younger than 7 years were included in the sample, we used the 85% threshold for the age-normalised reference values. This threshold was used to compare the sensitivity of each approach to predict the motor outcome in patients after rehabilitation.

### Statistical analysis

Statistical analyses were performed using the SPSS software (version 20, IBM Corp., Armonk, NY, USA). A Shapiro-Wilk test was performed to test for normality of the FA and NT values and WeeFIM motor outcome scores.

For the first aim, we compared the FA values derived from the ipsilesional CST between the three methods (atlas-based, streamline tractography, and the combined approach). Specifically, we calculated non-parametric Spearman rank correlation and determined differences between the three approaches using the Friedman test, followed by pairwise comparisons using the Wilcoxon signed-rank test. While the α error was generally set at 0.05, for these latter pairwise comparisons, we corrected for multiple comparisons using the Bonferroni correction. Additionally, Bland-Altman analysis was performed to define whether differences in approaches are clinically relevant.

For the second aim, we compared the relationship between the FA values and the NT with functional outcome at discharge of rehabilitation, by calculating the partial nonparametric correlation coefficient (ρ) between mean FA or NT and the WeeFIM motor scores, including age, rehabilitation time, and time of MRI scan after injury as covariates. The size of the correlation coefficient was defined for 0–0.25 as no or little relationship; 0.25–0.50 as a fair degree of relationship; 0.50–0.75 as a moderate to good relationship; and 0.75–1.00 as very good to excellent relationship [15]. Sub-analyses for each DTI protocol group (21 and 35 gradient directions) were performed. Since a range of TEs were used across the participant group, the relationship between FA and outcome was also tested after covarying for the TE (and for age, rehabilitation time, and the time of the MRI scan after injury). To test the regional specificity of the findings, an additional analysis using data from the contralesional and the ipsilesional CST was performed. This subgroup analysis was performed only in the patients who had experienced stroke (*n* = 20), since some of the patients with TBI demonstrated bilateral injuries.

Finally, for the tracts that were significantly associated with outcome in the non-parametric partial correlation analysis, we investigated the ability of the FA and NT parameters to differentiate between children with dependent versus independent functional outcome. We calculated the sensitivity, specificity, and area under the curve (AUC) of the DTI measures (mean FA and NT) using receiver operating characteristic (ROC) analyses. Additionally, the Youden index (J = sensitivity + specificity - 1) was calculated to determine the cut-off value of the DTI measures that could differentiate between the groups with the best combined sensitivity and specificity.

## Results

### Descriptive statistics

The Shapiro-Wilk test showed that the mean FA values for the atlas-based approach (*p* = 0.255; skewness -0.473, standard error (SE) = 0.378; and kurtosis -0.427, SE 0.741), the mean FA values for the combined approach (*p* = 0.061; skewness -0.626, SE = 0.378; and kurtosis -0.260, SE = 0.741), and the NT values for the streamline tractography approach (*p* = 0.237; skewness -0.075, SE = 0.378; and kurtosis -0.354, SE = 0.741) were normally distributed. The mean FA values from the streamline tractography approach (*p* = 0.004; skewness -0.968, SE = 0.378; and kurtosis 0.394, SE = 0.741), and the WeeFIM motor scores after rehabilitation were not normally distributed (*p* < 0.001; skewness -1.178, SE = 0.378).

### Comparison of FA values between the three approaches

**Fig. 3:**
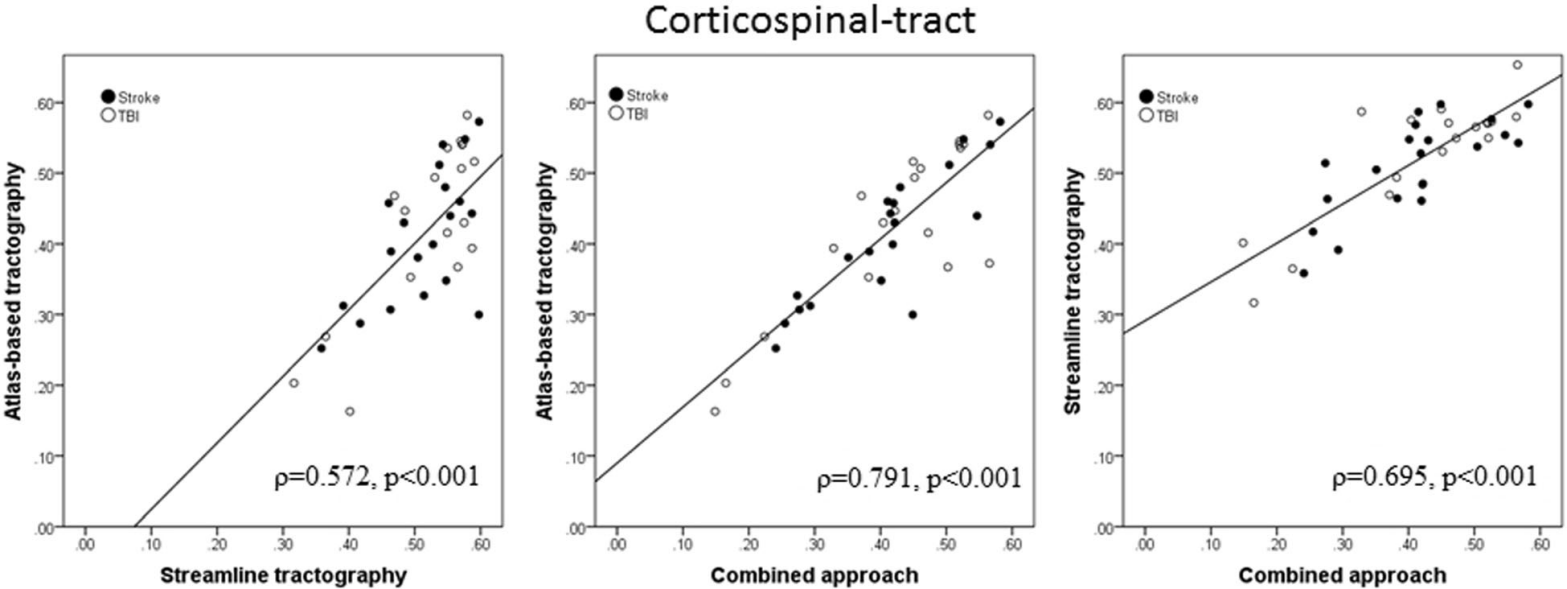
Scatterplot depicting the mean fractional anisotropy values measured with atlas-based, streamline tractography, and combined approach. Spearman rank correlation and injury (stroke and traumatic brain injury [TBI] included)

Table 3 includes the results of FA and NT values obtained using the three different approaches for each patient. Spearman rank correlation between mean FA values using the atlas-based and the streamline tractography approach was moderate (ρ = 0.572, *p* < 0.001; Fig. 3). Spearman rank correlation comparing the mean FA of the atlas-based and the combined approach was very good (ρ = 0.791, *p* < 0.001). Spearman rank correlation between mean FA values using the streamline tractography approach and the combined approach was good (ρ = 0.695, *p* < 0.001).

**Table 3:** Mean fractional anisotropy and number of tracts within the corticospinal tract in each patient derived from all approaches (atlas-based, streamline tractography, and combined)

Patients	Atlas-based	Streamline tractography		Combined
	mean FA	mean FA	NT	mean FA
1	.46	.57	1541	.41
2	.51	.54	1470	.50
3	.43	.48	1138	.42
4	.33	.51	1316	.27
5	.46	.46	482	.42
6	.48	.55	1231	.43
7	.44	.59	897	.42
8	.55	.58	1094	.53
9	.40	.53	1520	.42
10	.39	.46	886	.38
11	.38	.50	467	.35
12	.31	.39	53	.29
13	.31	.46	116	.28
14	.25	.36	5	.24
15	.30	.60	1182	.45
16	.54	.55	1379	.52
17	.47	.47	977	.37
18	.55	.57	740	.52
19	.54	.57	1490	.52
20	.58	.58	2154	.56
21	.49	.53	727	.45
22	.27	.36	440	.22
23	.20	.32	261	.17
24	.45	.49	1647	.42
25	.54	.57	1513	.53
26	.39	.59	1142	.33
27	.52	.59	2187	.45
28	.51	.57	2163	.46
29	.43	.58	1448	.40
30	.54	.54	814	.57
31	.35	.55	1105	.40
32	.29	.42	1299	.25
33	.57	.60	1442	.58
34	.44	.55	1407	.55
35	.37	.65	2109	.57
36	.37	.57	1334	.50
37	.35	.49	798	.38
38	.16	.40	190	.15
39	.42	.55	1010	.47

*FA* fractional anisotropy, *NT* number of tracts

The group analysis for different DTI protocols produced the following results. Spearman rank correlation in patients whose MRI scans were acquired using 21 gradient directions (*n* = 15) was very good between the atlas-based approach and the streamline approach (ρ = 0.782, *p* = 0.001), excellent between the atlas-based approach and the combined approach (ρ = 0.907, *p* < 0.001), and good between the streamline approach and the combined approach (ρ = 0.711, *p* = 0.003).

Spearman rank correlation in patients whose MRI scans were acquired using 35 gradient directions (*n* = 24) was fair but non-significant (ρ = 0.334, *p* = 0.111) between the atlas-based approach and the streamline approach, good between the atlas-based approach and the combined approach (ρ = 0.691, *p* < 0.001), and fair between the streamline approach and the combined approach (ρ = 0.411, *p* = 0.046).

The Friedman test showed a significant difference in FA values between different approaches (χ^2^ 57.282, *p* < 0.001). When performing pairwise comparisons (adjusted α level of 0.0167), we found that the streamline tractography approach had a significantly higher mean FA value (mean ± SD, 0.52 ± 0.08, range 0.32–0.65) than the atlas-based approach (0.42 ± 0.11, 0.16–0.58), and the combined approach (0.41 ± 0.11, 0.15–0.58). The Wilcoxon test showed significant differences between all approaches (*p* < 0.001).

**Fig. 4:**
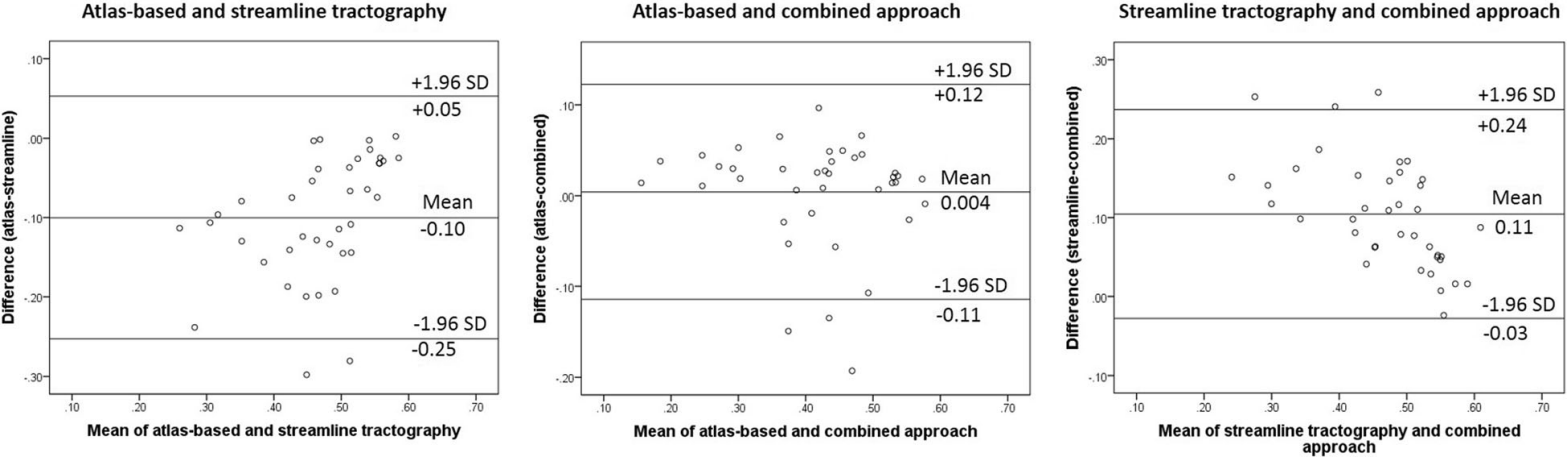
Bland-Altman plots showing the comparisons between approaches. *SD*, standard deviation

On Bland-Altman analysis (Fig. 4), the one-sample *t* test comparing FA values between atlas-based and streamline tractography, and between streamline tractography and the combined approach, showed significant differences (*p* < 0.001). The one-sample *t* test comparing FA values between the atlas-based and the combined approach was non-significant (*p* = 0.672). The linear regression coefficient for the difference in FA as the dependent variable and the mean as the independent variable using the atlas-based and the combined approach was non-significant (mean B -0.75, *p* = 0.424).

### Correlation with functional outcome

**Fig. 5:**
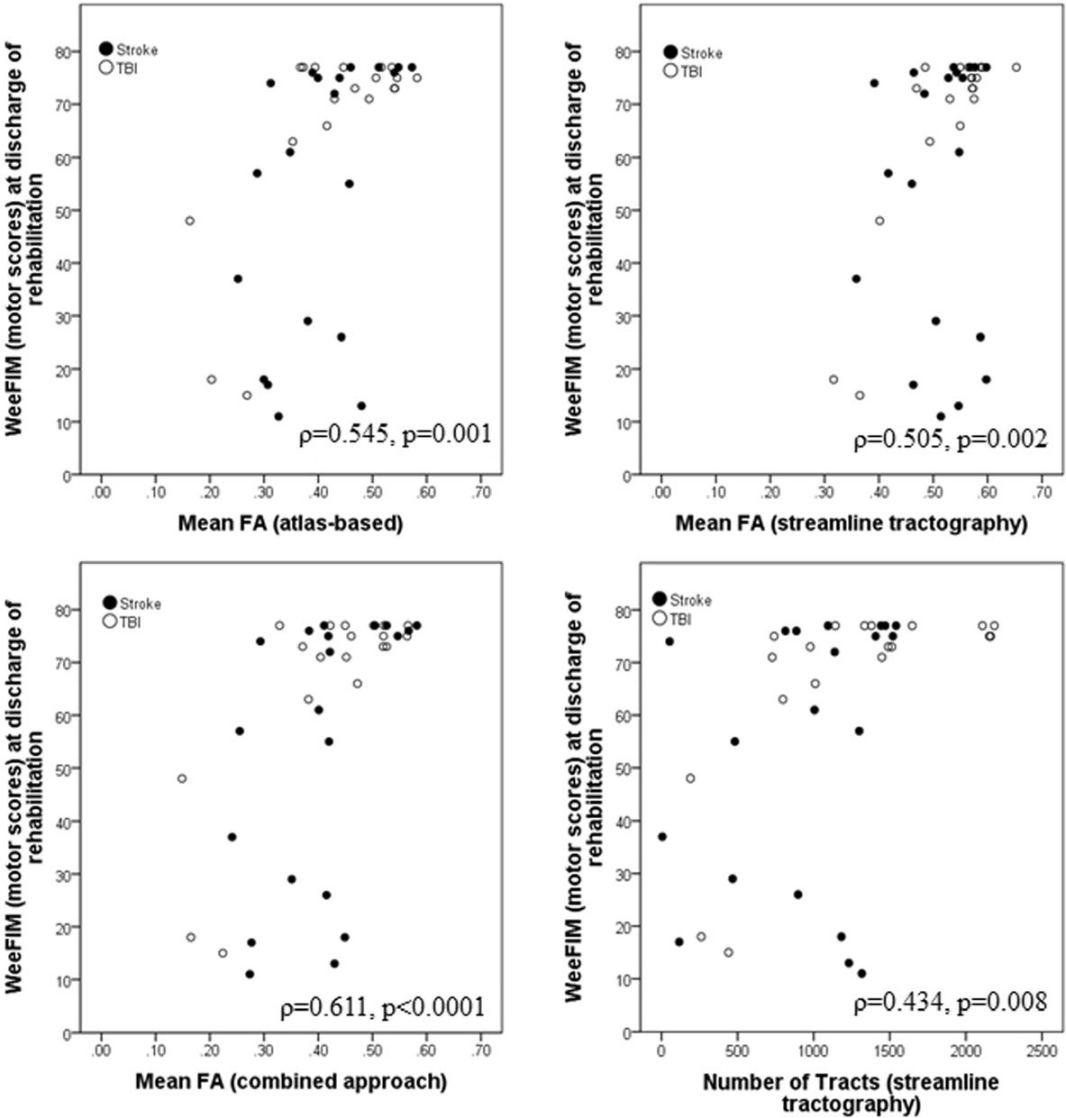
Scatterplots showing the relationship between the mean fractional anisotropy (FA) or the number of tracts and motor scores of the functional independence measure for children at discharge of rehabilitation for the different software methods for the corticospinal tracts. Nonparametric partial correlation analysis included age, rehabilitation time, and timing of magnetic resonance imaging after injury as covariates. *TBI*, traumatic brain injury; *WeeFIM*, functional independence measure for children

The nonparametric partial correlation analysis covarying for age, rehabilitation time, and timing of the MRI scan after injury showed significant moderate correlation between the WeeFIM motor outcome after rehabilitation and the mean FA from the ipsilesional CST for the atlas-based approach (ρ = 0.545, *p* = 0.001), the streamline approach (ρ = 0.505, *p* = 0.002), and the combined approach (ρ = 0.611, *p* < 0.001). These results were unchanged after including the TE as an additional covariate (*e.g.*, with the combined approach covarying for TE, ρ = 0.603, *p* < 0.001). Similarly, the analysis of nonparametric partial correlation revealed significant fair association (ρ = 0.434, *p* = 0.008; Fig. 5) between NT in the ipsilesional CST and WeeFIM motor outcome after rehabilitation.

The nonparametric partial correlation between the ipsilesional mean FA and the WeeFIM motor outcome in patients whose MRI scans were acquired using 21 gradient directions (*n* = 15) showed non-significant association for the atlas-based approach (ρ = 0.404, *p* = 0.192), the streamline approach (ρ = 0.199, *p* = 0.535), and the combined approach (ρ = 0.438, *p* = 0.155).

The non-parametric partial correlation between the ipsilesional mean FA and the WeeFIM motor outcome in patients whose MRI scans were acquired using 35 gradient directions (*n* = 24) showed significant fair to moderate correlation for the atlas-based approach (ρ = 0.488, *p* = 0.040), and the streamline approach (ρ = 0.583, *p* = 0.011). The combined approach showed a tendency towards positive correlation between FA and outcome (ρ = 0.466, *p* = 0.051).

The non-parametric partial correlation when calculated only in patients who had experienced stroke (*n* = 20) showed significant correlation between the WeeFIM motor outcome and the ipsilesional FA (ρ = 0.643, *p* = 0.005) and non-significant correlation between the WeeFIM motor outcome and the contralesional FA (ρ = 0.260, *p* = 0.313), derived using the combined approach.

### ROC analysis

Applying the threshold of 85% to classify clinical outcome scores into independent and dependent groups (good outcome, *n* = 30; poor outcome, *n* = 9), the ROC analysis demonstrated that the mean FA of the ipsilesional CST from the atlas-based analysis (sensitivity 90%, specificity 67%, Youden index 0.57, AUC 0.82), and the mean FA of the combined approach (87%, 67%, 0.53, 0.82) provided the highest combined sensitivity and specificity for outcome, in comparison to the NT from the streamline tractography method (50%, 100%, 0.50, 0.79), and the mean FA of the streamline tractography method (70%, 67%, 0.37, 0.67, respectively).

## Discussion

In the present study, we compared an atlas-based approach, a streamline tractography analysis approach, and a combined approach applied to the same DTI data to examine the relative accuracy of each method for predicting outcome in children with ABI, and to evaluate the advantages and disadvantages of each method (and their pre-processing approaches) for possible clinical use. We investigated the agreement between each method and the accuracy of each method for predicting clinical outcome using FA data from the CST.

For the primary aim of this study, we found differences between the FA values derived using the different approaches. For the second aim of this study, we found that the FA values from the ipsilesional CST calculated using both approaches (and the NT from streamline tractography) are significantly associated with the motor outcome after rehabilitation, but the atlas-based approach and combined approach incorporating an extra motion correction step had higher predictive accuracy for motor outcome, as assessed by ROC analysis.

One advantage of both streamline tractography and probabilistic tractography methods over atlas-based approaches is that they can be applied in native space, without registration to a template. While image registration and normalisation methods have demonstrated high reliability and accuracy in healthy brains [16], registration methods can provide inappropriate solutions when applied to images from patients with stroke or other brain injuries, where lesioned brains are matched to a template with no brain lesions [17, 18]. However, while atlas-based analyses can be automated to a large extent, streamline tractography results are dependent on manual delineations of the seed and target regions, so the results are arguably more operator-dependent than those from atlas-based analyses. In addition, streamline tractography methods are less accurate in cases where the raw DTI images were collected with a non-isotropic voxel resolution. Clinical DTI protocols like those utilised in the present study with a slice thickness (3 mm) considerably greater than the reconstructed in-plane resolution (0.94 × 0.94 mm) may, therefore, be less suited to streamline tractography analyses than protocols with an isotropic voxel resolution [19].

Operator dependence is also an important consideration for (non-tractographic) ROI analyses of DTI data. For example, Lilja et al. [20] showed that in quantitative DTI analyses of the optical tracts, results differ according to which ROI method (manual or semi-automatic) is applied. Similarly, Foeling et al. [21] reported high operator dependence associated with ROI analyses, after considering the importance of various factors like the ROI definition, atlas-based analyses, effects of motion, registration, and spatial normalisation. In a comparison of voxel-based and manual ROI-based analyses of DTI data in children and young adults, Snook et al. [22] observed good correlation between the FA values derived with automated and manual methods. However, they noted differences between the two methods in sensitivity to age effects in certain brain regions, thought to be due to the effects of spatial normalisation and smoothing in the voxel-based analyses. Based on the apparent differences in results between the methods they concluded that both manual (ROI-based) and voxel-based analyses offer complementary insight into neurodevelopment.

In the present study, the FA values derived with the streamline tractography analysis were significantly higher than those from the atlas-based analysis, despite moderate correlation between these two measures. The lower FA values measured with the atlas-based approach may be due to the inclusion of parts of the CST where degeneration reduced the FA beneath the tracking threshold for streamline tractography. Alternatively, these lower FA values may be due to partial volume effects. In the case of ABI, it is difficult to separate between these two effects (of degeneration and partial volume), as degeneration of the tract would be expected to cause a loss of white matter volume and a reduction in the “number of tracts” in the region previously occupied by the tract prior to the injury. However, the higher predictive accuracy for outcome observed with the FA values from the atlas-based approach suggests that the atlas-based FA values are clinically relevant, even if the atlas region includes parts of the tract that have undergone degeneration, or where the FA falls below the tracking threshold.

Our data also showed a link between the NT from streamline tractography and the motor outcome after rehabilitation. However, since the NT is vulnerable to bias from both experimental and biological factors [23], the FA may be a more robust indicator of white matter integrity, particularly in the clinical setting where the signal-to-noise ratio may be suboptimal and experimental parameters (including the voxel resolution) may vary. The FA values from the atlas-based method also seem to capture both aspects of degeneration (loss of volume/tracts and a reduction of FA in the remaining tracts) in a single measure, while these aspects are quantified separately with the NT and FA from the streamline tractography approach. While some previous studies have reported dependence of FA on the TE of the DTI acquisition [24], in the present study the link between FA and outcome remained unchanged after controlling for TE, suggesting that TE variations are unlikely to bias the apparent link between FA and outcome.

The combined approach (which used identical pre-processing to the streamline tractography method, including a motion correction step and eddy current correction, tensor fitting, and calculation of the FA maps) improved the prediction and was comparably or slightly more accurate for outcome in comparison to the atlas-based approach. Motion correction could most likely bring additional improvement to the accuracy of DTI for outcome prediction in datasets demonstrating significant motion during the scan.

Although the FA in the ipsilesional CST seems to provide a robust predictor of motor outcome, by considering only a single tract it is not possible to confirm the specificity of these findings, or whether, for example, FA values in other tracts also might predict motor outcome. Therefore, in order to test the regional specificity of the findings, we also exported the mean FA for the contralesional CST using the combined approach, and repeated the nonparametric partial correlation testing of the contralesional FA versus outcome. This additional analysis was performed just in the subgroup of patients with stroke, since some of the patients with TBI demonstrated bilateral injuries, potentially affecting both the contralesional and ipsilesional motor tracts. We found significant correlation between the WeeFIM motor outcome and the ipsilesional FA in the stroke subgroup, which was not present in the contralesional CST, providing some support for the regional specificity of the ipsilesional CST for motor outcome.

Several limitations should be taken into account for this study. As discussed by Soares et al. [6], there are many different software packages and tools available to pre-process and analyse DTI data. In this study, we only compared two particular implementations (an atlas-based method using FSL and a streamline tractography method using ExploreDTI), which both included pre-processing, tensor estimation, and tract selection. However, other methods (*e.g.*, manual ROI analysis, voxel-based analyses, and probabilistic tractography) and software packages (*e.g.*, SPM, Freesurfer, BrainVoyager, DoDTI, DTIstudio, Camino, etc.) are available for DTI data analyses, which were not considered in the present study. In addition to the FA, there are also other DTI metrics like the mean diffusivity, axial diffusivity, and radial diffusivity [25], which were not considered. Further comparisons between analysis methods and software implementations and across DTI metrics would be needed to establish the relative advantages and disadvantages of each approach, and the relative sensitivity of each DTI metric to outcome.

Another limiting factor is the heterogeneity of the patient group and the relatively small sample size, which made it difficult to perform an analysis like the ROC analysis for each subgroup (stroke versus TBI) or each DTI protocol (21 versus 35 directions). For example, in the group of patients in whom the DTI was acquired with 35 directions, only three patients had a poor outcome, and within the TBI group only three patients had a poor outcome. The timing of the MRI measurement after the injury also varied across the patient group. Some patients were measured first in another hospital using another MRI scanner (1.5-T field strength, not used in this study), or with computed tomography. Nevertheless, all MRI data included in the present study were acquired before the rehabilitation therapy and the time period between injury and MRI was included as a covariate in the correlation analysis. This group variability could not be corrected due to the retrospective nature of this study, but future studies incorporating larger patient group sizes, or with a prospective design may be able to account for variability in outcome arising from differences in the type or timing of acquired brain injury, or in the scanning protocol.

In conclusion, for the primary aim of this study, we found differences between the FA values derived using the different approaches. For the second aim, in a clinical DTI sample of children with ABI, FA values from streamline tractography were higher than those from the atlas-based and the combined approach. FA values for the CST derived from an atlas-based approach and combined approach provide better predictive accuracy for clinical outcome than those derived from streamline tractography. Nevertheless, FA values from both methods provide significant predictors for clinical motor outcome. The combined approach utilising an additional motion correction step seems to improve the accuracy of DTI as a predictor of the rehabilitation outcome.

## Abbreviations

ABI: Acquired brain injuries
AUC: Area under the curve
CST: Corticospinal tracts
DTI: Diffusion tensor imaging
EPI: Echo-planar imaging
FA: Fractional anisotropy
FSL: FMRIB software library
MNI: Montreal Neurological Institute
MRI: Magnetic resonance imaging
NT: Number of tracts
ROC: Receiver operating characteristic
ROI: Region of interest
SD: Standard deviation
SE: Standard error
TBI: Traumatic brain injury
TE: Echo time
TR: Repetition time
WeeFIM: Functional independence measure for children
